# Parity and dementia risk: Investigating the associations of number of biological children and cognitive and behavioural impairment in a Canadian sample

**DOI:** 10.1002/alz70857_096402

**Published:** 2025-12-24

**Authors:** Jasper F.E.U. Crockford, Dylan X. Guan, Maryam Ghahremani, Gillian Einstein, Cindy K Barha, Eric E. Smith, Zahinoor Ismail

**Affiliations:** ^1^ Hotchkiss Brain Institute, University of Calgary, Calgary, AB, Canada; ^2^ University of Toronto, Toronto, ON, Canada; ^3^ Rotman Research Institute, Toronto, ON, Canada; ^4^ Hotchkiss Brain Institute, Calgary, AB, Canada; ^5^ University of Calgary, Calgary, AB, Canada; ^6^ Department of Clinical Neurosciences, University of Calgary, Calgary, AB, Canada

## Abstract

**Background:**

The number of successful pregnancies (i.e., parity), is a sex‐specific factor that may influence susceptibility to cognitive decline and dementia. Evidence suggests a possible non‐linear relationship between parity and risk for cognitive decline, although findings are inconsistent. Beyond well‐appreciated later‐life cognitive changes, later‐life behavioural and personality changes, i.e., mild behavioral impairment (MBI), are also linked to greater dementia risk. However, the relationship between pregnancy history, cognition, and MBI symptoms remains unexplored. This study investigated these associations.

**Method:**

Participant data were from the Canadian Platform for Research Online to Investigate Health, Quality of Life, Cognition, Behaviour, Function, and Caregiving in Aging (CAN‐PROTECT) study. The cohort comprised 1,946 females (mean age 63.1±9.0), with 1,477 reporting at least one biological child (range of children = 1‐5+; nulliparity=469). Number of completed pregnancies were operationalized as the number of biological children. Cognition was assessed using the Revised Everyday Cognition (ECog‐II) scale. MBI symptoms were measured with the Mild Behavioral Impairment Checklist (MBI‐C). Higher ECog‐II and MBI‐C scores both indicate greater severity. Negative‐binomial regression models examined the associations between the number of biological children and ECog‐II and MBI‐C scores. Secondary analyses compared females with at least one biological child to those with none. All models were adjusted for age, education, ethnic origin, and age at first childbirth.

**Results:**

Having more biological children was associated with lower ECog‐II scores (b=‐8.42, 95%CI [‐14.01, ‐2.45], *p* = .058). While participants with more biological children also tended to have lower MBI‐C scores, with a similar magnitude and direction of effect, this association was not statistically significant (b=‐6.97, 95%CI [‐14.31, 0.99], *p* = .08). In contrast, having at least one biological child (versus none) was not significantly associated with ECog‐II (b=‐0.25, 95%CI [‐9.578, 9.91], *p* = .96), but was linked with lower MBI‐C scores (b=‐18.37, 95%CI [‐28.54, ‐6.74], *p* = .002), with a great difference in effect size between cognition and behaviour.

**Conclusion:**

Parity number was associated with better cognition and trended toward lower MBI symptom severity. Parity status was linked to lower MBI symptom severity but not fewer cognitive symptoms. These findings suggest that both parity status and number of biological children may affect later‐life cognitive and behavioural health.

